# Risk assessment of patients with diabetes for foot ulcers according to risk classification consensus of International Working Group on Diabetic Foot (IWGDF)

**DOI:** 10.12669/pjms.293.3473

**Published:** 2013

**Authors:** Hajieh Shahbazian, Leila Yazdanpanah, Seyed Mahmuod Latifi

**Affiliations:** 1 Hajieh Shahbazian, Professor, Endocrinologist, Diabetes Research Center , Ahvaz Jundishapur University of Medical Sciences, Ahvaz, Iran.; 2 Leila Yazdanpanah, MD, Diabetes Research Center , Ahvaz Jundishapur University of Medical Sciences, Ahvaz, Iran.; 3 Seyed Mahmuod Latifi, Msc in Biostatistics, Diabetes Research Center , Ahvaz Jundishapur University of Medical Sciences, Ahvaz, Iran.

**Keywords:** Diabetic foot, Diabetic neuropathy, monofilaments, ABI, Diabetic foot, IWGDF criteria

## Abstract

***Objective: ***The aim was assessment of diabetic foot ulcer risk factors according to International Working Group on the Diabetic Foot (IWGDF) consensus.

***Methodology: ***All referred patients with diabetes were divided into four groups based on IWGDF criteria (without neuropathy, with neuropathy, neuropathy with deformity or vascular disorders, foot ulcer or amputation history).

***Results: ***Mean age of patients was 53.8±10.7 years. Two hundred and sixty nine patients ​(62/6%) were female and 161(37/4%) were male. Twenty three percent had disturbed sense of vibration, 26% had decreased sensitivity to monofilaments and 17% had decreased pain sensation. Ankle brachial index (ABI) was abnormal in 6%. About 7% had history of prior ulcer. Patients were classified into four risk groups according to IWGDF criteria. Two hundred and seventy seven patients (65%) were in group 0, 75(17%) in group 1, 47 (11%) in group 2 and 31 (7%) in group 3. Patients in higher–risk groups had higher age, longer diabetes duration, higher HbA1C and less training (*p=0.0001, 0.001, 0.0001, 0.021 *respectively). The risk was higher in the presence of retinopathy (*p=0.005*). Patient's sex, BMI, smoking and nephropathy did not have significant correlation with risk of diabetic foot ulcer.

***Conclusion: ***This study showed that increase of age, duration of diabetes and HbA1c, lack of training and presence of retinopathy increases the risk of diabetic foot ulcers.

## INTRODUCTION

Diabetes is one of the main problems in health systems in the world.^1^ The world prevalence of diabetes among adults was 6.4%, and will increase to 7.7% by 2030.^2^

Patients with diabetes are at greater risk of complications, the most important of them are diabetic neuropathy^3^and peripheral vascular disorders^4^ that lead to diabetic foot ulcers.Currently the most common cause of neuropathy in western countries is diabetes.^3^ Diabetic neuropathy will develop in 50% of type 1 and 2 patients with diabetes.^3^^-^^6^ Diabetic foot problems are the most common cause of hospitalization in patients with diabetes^4^ and it accounts for 2 million patients with diabetes in the United States annually^7^ and often need long-term hospital admission.^8^ Diabetes is a major factor in half of all lower extremity amputations.^4^^, ^^9^

Diabetic foot ulcers occur in 15% of patients with diabetes in their life time.^4^^,^^9^^,^^10^ Risk factors for foot ulcer include male gender, duration of diabetes more than 10 years, peripheral neuropathy, foot deformity, peripheral vascular disease, smoking, history of prior ulcers or amputation, poor glycemic control,^1^^,^^6^^,^^10^ genetic and nutritional factors,^3^ diabetic retinopathy and nephropathy.^6^ Among them the main factor is peripheral neuropathy.^7^ The best approach in dealing with diabetic foot is prevention of ulcer through the identification of individuals at risk, patient education and follow-up.^4^ It is possible through routine foot exam, including previous history of the patient, the overall look, neurologic assessment (using 10 grams monofilaments and one of these examinations: 128 Hz tuning fork, pin prick, ankle reflexes)and vascular assessment (pulse palpation and measuring Ankle Brachial Index(ABI)).^6^^,^^11^

In the studies that have been performed in Iran, prevalence of neuropathy has been reported as 28.6-38%.^12^^,^^13^ Factors such as age over 50 years, diabetes duration more than 10 years, fasting blood sugar above 200 mg/dl, level of education, and deformity were risk factors of neuropathy; and 70% of patients were classified as high risk for developing diabetic foot ulcer according to IWGDF criteria.^12^ Similar studies have not been previously done in this region.

The purpose of this study was to assess risk factors for foot ulceration in patients with diabetes and the risk classification according to IWGDF (International Working Group on the Diabetic Foot) consensus^14^ in one of the reference diabetes centers in western south of Iran.

## METHODOLOGY

In this descriptive analytical study all patients with diabetes under 65 years referred to the diabetes clinic in Golestan Hospital, Ahvaz Jundishapur University of Medical Science from April to November 2011 were studied. Exclusion criteria of the study were hypothyroidism, pernicious anemia, discopathy, malignancy because they can also lead to neuropathy, and lower limb edema and congestive heart failure, because they can interfere with the assessment of neuropathy in examination and duration of diabetes less than 5 years in patients with type I because in this period neuropathy has still not developed.

Written consent was obtained from all patients and the study was approved by the Ethics Committee of Ahvaz Jundishapur University of Medical Science. A questionnaire including age, sex, BMI, diabetes duration, type of treatment, HbA1C, deformity, neuropathy symptoms, vascular symptoms, history of foot ulcer, previous training regarding foot care, smoking, history of retinopathy and nephropathy was completed for all patients. The patients were evaluated for deformity: contractured toe, prominent metatarsal heads and Halux valgus. Questions regarding symptoms of neuropathy and vascular disorder including numbness and tingling of toes and legs, pain and feeling hot or cold sensation in the legs, intermittent claudication, rest pain, thin skin, glossy and bluish skin discoloration and foot ulcer or amputation were asked from the patients.

Participant's feet were evaluated for callus and ulcer. The neurological examination was performed by 10 grams monofilament (superficial pressure), nourothesiometer (vibration perception), needle (superficial pain) and hammer (Achilles reflex).Superficial pressure was assessed by 10g monofilament. Patients closed their eyes while being tested. Nylon monofilaments were constructed to buckle to 90° angle. Four sites (1^st^, 3rd and 5^th^ metatarsal heads and plantar surface of distal hallux) were tested on each foot. Areas of callus, ulcer, scar and necrotic tissue were avoided in testing. Loss of the ability to detect this pressure at one or more sites on the plantar surface of the foot was considered as neuropathy.

Quantetive vibration perception was evaluated by Horwell nourothesiometer that was made in England. We put the probe on the bone of both toes and then regulated the vibration from low to high voltage up to the sensation of vibration by the patient. When the patient sensed and announced vibration, we checked the voltage and according to IWGDF criteria if it was more than 25 volts, it was reported as abnormal. Vibration perception above 25 Volts or decreased sensivity to 10 g monofilament was defined as neuropathy. For vascular examination dorsali spedis and tibialis posterior pulse were assessed. Then ABI (Ankle brachial index) was measured by hand held doppler device (HuntLighDiabetic Foot Kit) that was made in England, by evaluation of the flow signals from both arteries.ABI was calculated by this formula: 

ABI = (maximum systolic pressure of dorsalis pedis artery or tibialis posterior) / (maximum systolic pressure of brachial artery).

According to IWGDF consensus, ABI = 0.9-1.2 was considered as normal, ABI = 0.5-0.9as vascular disease, ABI<0.5 as severe vascular disease. Toe pressure was measured with the previous method and equal or less than 50 mm Hg was considered as vascular disorder. Patients were classified into four risk groups based on the presence of risk factors according to the consensus of IWGDF:

• Group 0: patients who had no distal sensory neuropathy 

• Group 1: patients who had only distal sensory neuropathy 

• Group 2: neuropathic patients who had foot deformity or vascular foot disease 

• Group 3: neuropathic Patients who had a history of prior foot ulcer or amputation

Study data were analyzed by spss19 and *P <0.05* was considered significant. To compare findings between groups analysis of variance (ANOVA), independent T and Chi Square test were used.

## RESULTS

In this study, 430 patients were examined of which 269(62.6%) were female and 161 (37.4%) were male. Mean age was 53.8 ± 10.7 years. Demographic characteristics of studied population is shown in Table-I. The mean duration of diabetes in the studied patients was 8.1 ± 6.6 years. Two hundred and sixty four (61%) of them complained of neuropathy symptoms (Table-II) and 7(2%) complained of vascular symptoms. Thirty one participants (7%) had prior history of foot ulcers and 131 (31%) had received previous training for foot care. Eighty one (20%) of patients had foot deformity (Table-II). On physical examination dry foot skin(19%) had the highest and callus (3%) had the lowest frequency. Retinopathy was present in 102 (24%) of patients and nephropathy in 87 (23%). Mean HbA1C was 8±1.8%. The overall prevalence of distal sensory neuropathy was 35% and vascular disease was 6%. Toe pressure was abnormal in 3% of patients. Patients were classified into four risk groups based on the presence of risk factors according to the consensus of the International Working Group on the Diabetic Foot(IWGDF):

• Group 0: 277cases(65%) • Group 1: 75cases (17%) • Group 2:47cases (11%) • Group 3: 31 cases (7%)

Foot ulcer correlated factors such as age, sex, BMI, diabetes duration, presence of previous training regarding foot care, smoking, retinopathy, nephropathy are shown in Table-III. After Anova and chi square test in all groups, the result showed that age of patient and diabetes duration increases the risk of foot ulceration significantly (*p=0.0001, 0.001* respectively).Previous training in foot care was significantly lower in high-risk groups (*P=0.021*). The patient's HbA1c levels were higher in high risk groups *(p=0.0001*). Retinopathy was present more in high- risk groups significantly (*P=0.005*).The patient's sex, BMI, history of smoking and nephropathy did not have significant correlation with higher risk groups (*p=0.08, 0.2, 0.5, 0.05* respectively).

We used logistic regression analysis to compare some variables(age, sex, type of treatment, diabetes duration, BMI, HbA1c, foot deformity, prior training regarding foot care, history of smoking, retinopathy, nephropathy and ABI) with history of diabetic foot ulceration in patients. Among them there was significant correlation between history of diabetic foot ulcer with HbA1c (OR=1.49 ci 1.17-1.90 &p=0.001), patient's previous training (OR=4.4 ci 1.19-16.24&p=0.026), vibration perception above 25 volts (OR=9.36 ci 3.04-28.78&p=0.00001) and decreased 10g monofilament sensation (OR=1.78 ci 1.04-3.05&p=0.035).

## DISCUSSION

In this descriptive analytical study, patients were evaluated for diabetic foot ulcer risk factors and classified based on IWGDF criteria into four groups: 65% of patients were in group 0, 17% in group 1, 11% in group 2 and 7% in group 3. Patient's age, diabetes duration, HbA1c, lack of previous training about foot care and retinopathy increased risk of diabetic foot ulcers significantly but patient's sex, BMI, smoking and nephropathy did not have significant correlation with risk of diabetic foot ulcer. In this study, the prevalence of distal sensory neuropathy was 35% that was between 23 to 42% in other studies^12^^,^^15^^-^^17^ which is comparable with our study. In this study patient's age and duration of diabetes and HbA1c and lack of previous training about foot care and presence of retinopathy increased the risk of diabetic foot. In other studies age, HbA1c, duration of diabetes, male gender increased the risk of diabetic foot.^12^^,^^18^ This study showed no significant correlation with sex. It may be because of a larger female population in our study.

**Table-I:** Demographic characteristics of study participants

Demographic Data	Number	Percent (%)
Number of patients	430	
Gender:		
Female	269	62.6
Male	161	37.4
Treatment:		
Oral Hypoglycemic agent	336	78
Insulin	94	22
Diabetes duration (Year):		
<5	166	39
5-10	149	34
10-15	55	13
15-20	42	10
>20	18	4
BMI( kg/m2 ):		
≤18.5	5	1
18.6-24.9	90	21
25-29.9	202	47
≥30	133	31
HbA1C(%):		
≤7	125	29
7.1-7.9	94	22
≥8	211	49
History of smoking	28	6
Retinopathy:		
No DR	328	76
NPDR	79	19
PDR	23	5
Nephropathy:		
No	332	77
Microalbuminuria	65	15
Overt proteinuria	32	8

*NPDR=non proliferative diabetic retinopathy **PDR=proliferative diabetic retinopathy

**Table-II:** Neuropathy symptoms and foot deformities frequency in study participants

	*n*	*%*
Neuropathy symptoms		
Tingling	42	10
Numbness	10	2
Sharp pain	6	1
Hot or cold sensation in foot	22	5
More than one symptom	184	43
Without symptom	166	39
*Foot deformity*		
Contractured toe	4	1
Prominent metatarsal head	60	14
Hallux valgus	9	2
More than one deformity	8	2

**Table-III:** Comparison of study variables based on IWGDF classification

	*Group0* *277 cases* *(65%)*	*Group1* *75 cases* *(17%)*	*Group2* *47 cases* *(11%)*	*Group3* *31 cases* *(7%)*	*P value*
Age(year)	52±11.3	56±9.5	58±7.5	55±8.9	0.0001
Sex(male/female)	97/180	30/45	16/31	18/13	0.08
BMI(kg/m2)					
≤18.5 18.6-24.9 25-29.9 30≤	4(1%) 59(21%) 132(48%) 82(30%)	0(0%) 10(13%) 35(47%) 30(40%)	1 (2%) 11(23%) 23(49%) 12(26%)	0(0%) 10(32%) 12(39%) 9(29%)	0.2
Diabetes duration(year)	7.2±6.2	9.5±6.7	9.6±7.5	11±7.4	0.001
Previous patient training about foot care	96(35%)	22(29%)	9(19%)	4(13%)	0.021
HbA1c (%)	7.9±1.7	8.2±1.8	7.7±2	9.5±1.8	0.0001
Smoking	16(6%)	5(7%)	1(2%)	6(19%)	0.5
Retinopathy	51(18%)	25(33%)	14(30%)	12(39%)	0.005
Nephropathy	56(20%)	16(21%)	12(26%)	13(42%)	0.05

In one study high plantar pressure, previous history of foot ulcer, duration of diabetes, deformity and male gender was associated with increased risk of foot ulcer.^19^ In another study the relationship between smoking and foot ulcer was shown^1^ but this relationship was not found in this study. In classification of patients in risk groups according to IWGDF in other studies the prevalence of each group has been reported as: group 0 (30 -57%), group 1 (10- 29%), group 2 (16-27%) and group 3 (14-17%).^12^^,^^15^ It seems that our patients are in lower risk groups than other studies.

To identify vascular disorders, the simplest way is to palpate peripheral pulses, but another examination is using a hand held Doppler to determine ankle brachial index (ABI).In this study peripheral pulses were palpable in all patients and no vascular symptoms such as intermittent claudication or loss of leg hair was seen but the frequency of abnormal ABI was 6% (26 cases). In other studies, abnormal ABI have been reported 12-21%^15^^, ^^17^which has been associated with male gender, but in our study, sex showed no significant relationship with vascular disorders (p=0.6).In our study the prevalence of abnormal ABI was lower than other studies. Previous history of foot ulcers in studied patients was 7% that in comparison with other studies (5-16%) was in low prevalence range.^12^^, ^^15^^-^^17^

Our patients were in lower risk groups than other studies and they had lower prevalence of abnormal ABI and previous history of foot ulcer. It may be because of differences in age, diabetes duration, HbA1c, level of previous training and genetic factors in studied patients in comparison with other studies. For reducing the prevalence of diabetic foot ulcer, follow-up of the patients in group 0 annually, group 1 every 3 to 6 months, group 2 every 2 to 3 months and group 3 every 1 to 2 months is necessary.^6^

The practical value of this study is that it was the first study about risk assessment and classification of diabetic foot in this region and its findings can be useful in prevention and management of diabetic foot and consequently may reduce the burden of this complication. The limitations of this study were that the evaluation of patients was performed in a public diabetes clinic and some of the patients with diabetic foot ulcer may be referred to a special foot clinic therefore it may affect our data. In this study, patients were not separated by the type of diabetes. Rate of foot ulcer may be different in each type. This study's documented contents can be significant for regional healthcare policy makers because it can show the rate of neurologic and vascular involvement in feet of patients with diabetes, the importance of educating patients in the prevention of diabetic foot and remind that performing screening examinations can prevent such effects. We suggest further studies to evaluate the effectiveness of reducing risk factors to prevent diabetic foot ulcer and study the relationship of other risk factors such as footwear and plantar pressure for foot ulceration.

## CONCLUSION

This study shows that increasing age, duration of diabetes, HbA1c, lack of previous education and retinopathy increases the risk of diabetic foot. Gender, smoking, BMI and nephropathy were not associated with increased risk of foot ulcer. There was significant correlation between history of diabetic foot ulcer with HbA1c, patient's prior training, vibration perception above 25 volts and decreased 10g monofilament sensation.
